# Development and validation of a military fear avoidance questionnaire

**DOI:** 10.3389/fresc.2022.979776

**Published:** 2022-10-03

**Authors:** Carly Cooper, Bruce Frey, Charles Day

**Affiliations:** ^1^ Brooke Army Medical Center, Department of Occupational Therapy, US Army, Fort Sam Houston, TX, United States; ^2^ Department of Educational Psychology and Research, University of Kansas, Lawrence, KS, United States; ^3^ Irwin Army Community Hospital, Soldier Centered Medical Homes, US Army, Fort Riley, KS, United States

**Keywords:** fear-avoidance, questionnaire, military, pain, kinesiophobia, musculoskeletal

## Abstract

Chronic pain due to musculoskeletal injury is one of the leading causes of disability and reduced combat readiness in the U.S. Army. Unidimensional pain management systems are not effective in addressing the complex phenomenon of pain-related disability. Growing evidence has supported use of the Fear Avoidance Model (FAM) as a suitable model to address pain-related disability and chronicity from a multidimensional pain neuroscience approach. While several fear avoidance measurement tools exist, one that addresses the complexity of the Army environment encouraged the authors to develop and test the reliability and validity of a military specific questionnaire. This study developed and validated an Army specific fear avoidance screening, the Return to Duty Readiness Questionnaire (RDRQ), which subsequently demonstrated good psychometric properties. Reliability coefficients demonstrate high internal consistency values both during pilot study (*α* = 0.96) and validation study (*α* = 0.94, *ωt* = 0.94). A Correlation Coefficient of 0.74 when compared with the Fear Avoidance Components Scale (FACS) suggests good concurrent validity. Future study should include replication in a new army population, investigation of responsiveness, test-retest reliability, structural validity and establishing severity scores with minimal clinically important differences to enhance utility.

## Introduction

Chronic pain stemming from musculoskeletal injury can be devastating both physically and psychologically. For the individual, the physical and psychological effects of injury and associated chronic pain, pain continuing for longer than 12 weeks despite treatment, impacts all areas of their life, from work to parenting, to occupations of daily living (1–3). At an institutional level, the effect of injury and subsequent chronic pain problems is costly both monetarily and through lost productivity (4–6). This challenge is no different for the U.S. military and the men and women who serve. The Army is contending with staggering numbers effecting force readiness explicitly related to musculoskeletal injury. A holistic health movement in Army medicine postures that the traditional unidimensional way of managing and preventing chronic pain has been largely unsuccessful. As a result, the Army has shifted to a health initiative that makes educative, nonpharmacological, and holistic approaches to pain management a priority in preventing pain- related disability (7).

Research investigating pain phenomena has found evidence linking fear avoidance behaviors with pain-related disability, and that incorporating fear avoidance screening and assessment is useful for successful evidence-based, multidisciplinary treatment planning (8–11). One model of chronic pain that has been significantly researched is the Fear Avoidance Model (FAM) (12, 13). This specific model has been found to be associated with pain-related disability in a military population, encouraging the use of fear avoidance behavior screening in military musculoskeletal injury management (14, 15). Pain is a complex phenomenon which has spurred the realization that unidimensional metrics being used to define pain are unproductive and can lead to ineffective treatment strategies (14). This concern has led to the use of more holistic self-report measures assessing pain perception in addition to physical and psychological detriments because of pain. The FAM has gained in popularity since its initial inception as a multidimensional model to explain the development of chronic pain and association with pain-related disability (16). This has directed to the development of various self-report health questionnaires for worker's compensation and athletic population to understand the psychosocial aspect of injury recovery, but none exist that encompass the complexities and vocabulary of the military environment (17–20). Literature suggests that a military specific version of a fear avoidance questionnaire will be a valuable tool for Army primary care and rehabilitation providers (14). Measurement tools that incorporate the theoretical components of the FAM are few and have been validated on non-military populations. In response to this, an Army specific fear avoidance screening tool, the Return to Duty Readiness Questionnaire, was developed to capture the complexities of the military environment and the effects of injury on the soldier regarding fear avoidance behaviors (21). The objective of this study was to develop and test the reliability and validity the Return to Duty Readiness Questionnaire (RDRQ) through analysis of internal consistency and establishing content and concurrent validity.

## Methods

### Questionnaire development and pilot study

Experts in the warrior care and recovery field contributed to the development of the scale throughout November 2019. We used a modified method of scale development starting with contributions from experts, similar to the methods used to create the Athlete Fear Avoidance Questionnaire (AFAQ) (17, 21). Initially a panel of five experts gathered to contribute to item development and construction. Many items were adapted from existing validated fear avoidance questionnaires (17–20, 22, 23) to reflect the uniqueness of the military environment and vocabulary. The panel consisted of four experts including an Army Physician Assistant, Tactical Strength Coach, Physical Therapist, Occupational Therapist. Although each expert had experience in different areas, they all shared expertise working with return to duty optimization for soldiers who have been injured or experiencing pain. Panel participation was completed electronically. Background information about fear avoidance and the Fear Avoidance Model was discussed along with each member's perception of how that theory is relevant to the military population. All items generated by experts were gathered and sent to all panel members as a comprehensive list to provide an opportunity for revision and further refinement. Those same members along with an additional three experts from the field were asked to rate each item based on applicability to measuring fear avoidance in soldiers on a scale of 1 (not at all relevant) to 5 (very relevant). During this initial scale development phase, eight active-duty soldiers were asked to complete and study the pilot questionnaire. A semi-structed focus group was held with these soldiers where they were asked to give their opinion on the items and provide feedback on items they viewed as most/least relevant. Following three rounds of comments and ratings from the experts, combined with results from the focus group discussion, twenty-six (26) items remained as the original scale after 17 items were excluded due to non-relevance, or justification for removal. The final title, “Return to Duty Readiness Questionnaire” was agreed on during focus group discussion, as well as the addition of a comments section, in preparation for dissemination for pilot study.

Active-duty soldiers seeking primary care for musculoskeletal pain were asked to complete the RDRQ with appointment paperwork as part of a project improvement process at an Army installation soldier care clinic. No personally identifiable or health information was taken from the thirty participants as part of questionnaire completion. Cronbach's alpha is a widely used statistic used to demonstrate internal consistency of the instrument in question (23, 24). The concept that the sum of items in a scale are representative of measuring the same construct is an important part of determining the internal consistency of that measure. The statistic is determined using a formula that accounts for the scale length and the strength of correlations among the items of the scale, ranging from 0.00–1.00 with various interpretation results (25). This commonly used statistic was used to provide pilot scale reliability estimates reported in the results section (23).

### Questionnaire validation study

A new population of soldiers from the same Army installation as the pilot study were again asked to complete the RDRQ as part of their standard of care primary care paperwork when they were being seen at the soldier care clinic for musculoskeletal pain. Again, personally identifiable or health information was not collected in relation to the RDRQ. Completed questionnaires were collected by the clinic chief for hand over to the primary researcher for analysis. Data analysis was approved by all appropriate institution's board of ethics whose approval was granted before any research activity was initiated. All data were analyzed using R statistical analysis software with significance levels as applicable set at *p* = 0.05. All participants had completed the RDRQ, and a smaller subset of soldiers also completed the FACS questionnaires as part of their medical visit with the primary care provider at the soldier clinic (20, 22).

In addition to Cronbach's alpha coefficient to estimate internal consistency, McDonald's Omega is an alternative statistic for evaluating internal consistency that is said to be more appropriate for a multidimension scale (26, 27). McDonald's Omega incorporates the factor loadings as non-essentially tau equivalent making it different from Cronbach's alpha coefficient and assume all the items reflect a single common underlying variable making this a more realistically reflective view of total score reliability (24, 28). Both Cronbach's coefficient alpha, and McDonald's coefficient omega was estimated as a method of assessing the internal consistency of the RDRQ using the psych and sem/semTools packages (26, 29–31).

Many scale development and validation scale studies determine a correlation coefficient by correlating the scale in question to a related, established scale to establish concurrent validity (17–20). This process is important for showing that the scale reflects the intended construct. To verify validity of intended construct, the R psych package was used to calculate Spearman correlation coefficients between the total scores of the RDRQ and total scores of the completed FACS to establish concurrent validity as it has been established as a valid and reliable fear avoidance questionnaire (21, 32). The FACS is associated with self-reported disability, pain, and measures of endurance in response to pain (19). The FACS has been used to study chronic pain populations as part of a military Functional Restoration (20, 21).

## Results

All data were analyzed using R statistical analysis software with significance levels as applicable set at *p *= 0.05. RDRQ validity analysis was completed using R 4.1.1. Survey results were input by the researcher from completed questionnaires into a raw data excel spreadsheet for analysis.

### Questionnaire development and pilot study

A total of 32 RDRQ surveys were collected for initial analysis. Subjective data from focus group suggested questions numbers 1, 3, 8, 16, and 17 of the original questionnaire be removed. Question number 12 reworded to reflect more of a “what can I do” type of question, number 11 be reworded to reflect how pain is affecting others, and to combine items RDRQ14 and RDRQ15 as it was perceived to be redundant. There was also a unanimous suggestion to add a comments box at the end of the questionnaire. The title of “Return to Duty Readiness Questionnaire” was agreed upon during this time as well keeping in mind the perspective of the soldier if they were to see the words “fear avoidance” in the title of a questionnaire regarding their healthcare. The focus group's comments imply that they were enthusiastic about completing the survey and found it very interesting, suggesting that respondents completed the survey thoughtfully and did not reply randomly (24).

Analysis of this data showed a Cronbach's *α* coefficient of 0.94 for the initial pilot RDRQ, indicating high internal consistency (21). Statistical analysis did not indicate that removal of any item would increase the coefficient resulting in a final number of 20 items. Response options range from 1 (Not at All) to 5 (Completely Agree) which should allow for adequate variability to produce reliable results (21, 24).

### Questionnaire validation study

Data from 240 soldiers was analyzed for validation assessment of the final questionnaire established during pilot study. Internal consistency was established with a Cronbach *α* coefficient of 0.94 and McDonald's omega total coefficient of *ωt* = 0.96, and all items correlated with the total score (*α* > 0.52). Reliability estimates were also computed on the soldier responses to the FACS (*n* = 143), which resulted in similar estimates (*α* = 0.94, *ωt* = 0.95).

To establish concurrent validity evidence of the RDRQ in comparison to a known fear-avoidance scale standard, total scores were correlated with total scores from the FACS. Completed RDRQ and FACS instruments were collected from 158 soldiers in order to allow correlation coefficients to be calculated and relationship between the two scales to be analyzed using the R psych package cor.test function (32). Shapiro-Wilk tests of normality on the RDRQ and FACS total scores reveal non-normality. For this reason, Spearman's rank correlation coefficient was calculated as a non-parametric test of correlation. Spearman correlation coefficient results of this calculation show a significant correlation between the two sets of total scores (rho = 0.74, *p* < .001). This analysis indicates acceptable concurrent validity suggesting that the RDRQ is measuring the intended Fear-Avoidance construct.

## Discussion

### Questionnaire reliability and validity

During initial development, RDRQ items were developed using the FAM and other known fear avoidance instruments as a guide to address the unique military environment. This process resulted in the final 20 item questionnaire. Confidence in construct validity of the questionnaire generated is warranted as items were developed and examined by a panel of subject matter experts who were selected specifically to reflect the nature and intended utility of the scale (32). Content validity during pilot study were further evaluated with a focus group provided feedback from the patients found the RDRQ items relevant, appropriate, and understandable. High internal consistency coefficients during pilot study and validation study suggest that the RDRQ demonstrates good reliability. Of note, reliability analysis of the soldier responses to the FACS resulted in similar estimates.

Concurrent validity was established by the significant correlation between the RDRQ and the FACS, an existing validated fear avoidance tool (20, 21). A correlation coefficient of 0.74 rejects the null hypothesis but not as strongly as we would like, as the high value may suggest that the scales are measuring the same construct. This result, however, does indicate that the RDRQ accurately measures fear avoidance in this population. While the FACS has been used with a military population before, it hasn't officially been validated on an active-duty Army population (5, 14). The RDRQ was based on the same theoretical background as the FACS, but with a military-specific aspect, which is not present in the FACS (20, 21). It makes sense, then, that the RDRQ be highly correlated with the FACS without dejection of its ability to specifically measure military fear avoidance as they measure similar principles making the considerably high coefficient value acceptable.

### Limitations

Limitations of this scale development were that it did not include a pain measure to track whether a soldier's level of pain would affect the results. Other demographics such as gender, age, occupational specialty, and military rank may affect the results. Additional testing will be needed to compare pain-related fear in soldier's based on these demographics and should be accomplished with future studies. Further responsiveness validation is needed to correlate results on the RDRQ with return-to-duty time in injured soldiers. Concurrent validity was established using the Fear Avoidance Components Scale (FACS), which is an instrument that has been used with a military chronic pain population in a functional fitness arena but not officially validated on an active-duty Army population (5).

Also, a portion of this data was collected after a significant event change in the Army Combat Fitness Test (ACFT), which may have affected responses to scale items. Most of the questionnaires were collected during the worldwide COVID-19 pandemic, at which time the Army's new ACFT roll-out was put on hold. This rare situation could have also altered how soldiers responded to questions on either instrument as the definition of daily duties and soldier responsibilities changed in that timeframe.

### Future directions / recommendations

Larger sample sizes are required to conduct measurement invariance testing. This may offer additional insight into items that are more susceptible to certain rank groups or gender, providing further validity evidence based on internal structure.

The simple addition of a pain visual analog scale to the questionnaire in future study of responsiveness would provide valuable comparison evidence in terms of actual pain classification to fear avoidance behavior rating. Confirmatory factor analysis study can provide evidence of structural validity by confirming the dimensions of the FAM.

The Fear Avoidance Components Scale (FACS) was found to be reliable as measured by internal consistency with the sub sample that completed it in addition to the RDRQ. The FACS was developed for general painful conditions and while it has been used on military samples for research, it has not been officially validated for the military musculoskeletal pain population. Given the high concurrent validity between the RDRQ and FACS and the established internal consistency of the FACS in this sample, future studies may be done to establish validity of the FACS in military populations.

## Conclusion

This study suggests that the Return to Duty Readiness Questionnaire (RDRQ) measures factors of fear avoidance and can be used as a reliable fear avoidance questionnaire in Army musculoskeletal pain populations seen at the study site. Reliability estimates demonstrated good internal consistency of the RDRQ in this sample. Concurrent study with a test criterion provided concurrent validity evidence that for this sample the RDRQ is measuring fear-avoidance. This study should be replicated on other military samples to gain utility of use at additional installations. Future study should also include investigation of measurement invariance, responsiveness, confirmatory factor analysis to confirm structural validity, test-retest reliability, internal consistency of the FACS on a military population. The use of a fear avoidance questionnaire in this population will allow clinicians to detect patients that would benefit the most from other medical referrals or educational intervention as part of their rehabilitation process.

## Data Availability

The raw data supporting the conclusions of this article will be made available by the authors, without undue reservation.
